# Draft Genome Sequence of *Pseudomonas stutzeri* ODKF13, Isolated from Farmland Soil in Alvin, Texas

**DOI:** 10.1128/genomeA.00293-16

**Published:** 2016-04-14

**Authors:** Rupa Iyer, Ashish Damania

**Affiliations:** a Center for Life Sciences Technology, College of Technology, University of Houston, Houston, Texas, USA; b Department of Pediatrics, Section of Pediatric Tropical Medicine, Baylor College of Medicine, Houston, Texas, USA

## Abstract

*Pseudomonas stutzeri* ODKF13 is a bacterial microorganism isolated from farmland soil in Alvin, Texas. This strain is notable for its naphthalene degradation and nitrogen fixation pathways and for its characterization as an organophosphate degrader of phosphotriester and phosphorothioate insecticides.

## GENOME ANNOUNCEMENT

*Pseudomonas stutzeri* is a Gram-negative bacterium known for its high capacity for nitrogen fixation and opportunistic pathogenicity. In addition, several *P. stutzeri* strains have also been found to possess xenobiotic degradation capacity, particularly against naphthalene and other similar aromatics (1, 2). Here we report a draft genome sequence of a soilborne microorganism identified as a novel strain of *P. stutzeri* that was isolated from farmland in Alvin, Texas through an Environmental Sampling Research Module (3) undertaken by University of Houston biotechnology undergraduates. Sequence analysis of this new strain, designated *P. stutzeri* ODKF13, shows that it shares nitrogen metabolism pathways common to the species and is most closely related to *P. stutzeri* T13 and *P. stutzeri* B1SNM1, a strong nitrogen fixer and naphthalene degrader, respectively (2, 4). In addition, *P. stutzeri* ODKF13 has been characterized as a strong organophosphate degrader on both phosphotriester and phosphorothioate substrates. Taken together with its putative nitrogen-fixing capabilities, ODKF13 has great potential as a viable bioaugmentation strain in agriculture. The genome sequencing of ODKF13 was performed through Illumina MiSeq paired-end sequencing with a final sequencing coverage of 289×. Sequence reads were checked for quality using Fastqc (http://www.bioinformatics.babraham.ac.uk/projects/fastqc/) and filtered using BBTools (http://sourceforge.net/projects/bbmap). Paired-end reads were then assembled into a total of 85 contigs with the Spades 3.6.2 program (5). Preliminary reference based annotation using PATRIC (6) was carried out to identify conserved pathways. Final *de novo* annotation was performed with Prokka (7) and the NCBI Prokaryotic Genomes Automatic Annotation Pipeline (http://www.ncbi.nlm.nih.gov/genome/annotation_prok/). The metabolic pathways of aromatic and heterocyclic compounds were examined through KEGG databases (8). This draft genome of strain ODKF13 consists of a total of 4,591,328 bp encoding for 4,134 putative coding sequences, of which 3,336 are predicted to form functional proteins. The genome also contains 5 rRNA, 54 tRNA, and 4 noncoding RNA (ncRNAs) loci.

### Nucleotide sequence accession numbers.

The *P. stutzeri* ODKF13 whole-genome shotgun (WGS) project has the project accession no. LSVE00000000. This version of the project (01) has the accession number LSVE01000000, and consists of sequences LSVE01000001 to LSVE01000085.
